# Deep VMD-attention network for arrhythmia signal classification based on Hodgkin-Huxley model and multi-objective crayfish optimization algorithm

**DOI:** 10.1371/journal.pone.0321484

**Published:** 2025-05-14

**Authors:** Hang Zhao, Xiongfei Yin

**Affiliations:** School of Physics and Optoelectronic Engineering, Hangzhou Institute for Advanced Study, University of Chinese Academy of Sciences, Hangzhou, China; University of Queensland - Saint Lucia Campus: The University of Queensland, AUSTRALIA

## Abstract

Recent research for arrhythmia classification is increasingly based on AI-driven approaches, which are primarily grounded in ECG data, but often neglect the mathematical foundations of cardiac electrophysiology. A finite element model (FEM) of the human heart, grounded in the Hodgkin-Huxley (HH) model was established to simulate cardiac electrophysiology, and ECG signals from 200 representative points were acquired. Two types of arrhythmia characterized by significant anomalies in the variables of the HH model were simulated, and corresponding synthetic ECG signals were generated. A multi-objective optimization method based on non-dominated sorting was integrated into the crayfish optimization algorithm (MOCOA). To optimize the key parameters *K* and α in variational mode decomposition (VMD), a MOCOA-VMD technique specifically tailored for ECG signal processing was developed. The Pareto optimal front was generated using MOCOA with the indicators of spectral kurtosis and KL divergence, by which the optimal intrinsic mode functions were obtained. A deep VMD-attention network based on MOCOA was developed for ECG signal classification. The ablation study evaluated the effectiveness of the proposed signal decomposition method and deep attention modules. The model based on MOCOA-VMD achieves the highest accuracy of 94.46%, outperforming models constructed using EEMD, VMD, CNN and LSTM modules. Bayesian optimization was employed to fine-tune the hyperparameters and further enhance the performance of the deep model, with the best accuracy of the deep attention model after TPE optimization reaching 96.11%. Moreover, the real-world MIT-BIH arrhythmia database was utilized for further validation to prove the robustness and generalizability of the proposed model. The proposed deep VMD-attention modeling and classification strategy has shown significant promise and may offer valuable inspiration for other signal processing fields as well.

## Introduction

Cardiovascular disease (CVD) is a leading global cause of morbidity and mortality, responsible for more than 31% of worldwide deaths in 2020 [1]. Cardiac arrhythmias, defined as deviations from the normal heart rate or rhythm that cannot be explained by physiological factors [2], represent a significant subset of CVD. These arrhythmias can present as irregular heartbeats, excessively fast heart rates (tachycardia), or abnormally slow heart rates (bradycardia), which can be benign or indicate serious underlying heart conditions. Timely and precise identification of arrhythmias not only facilitates appropriate therapeutic interventions but also aids in preventing potential complications and improving patient outcomes [3].

The traditional rule-based approach to diagnosis often struggles with the complexity and heterogeneity of large-scale medical data, necessitating intensive analysis and substantial medical expertise to achieve reliable diagnostic outcomes. In recent years, the application of artificial intelligence (AI) in electrocardiogram (ECG) interpretation has become a transformative development within the cardiovascular domain. Leveraging vast repositories of digitized clinical ECG data, researchers have developed sophisticated AI models designed to identify various cardiac conditions, such as left ventricular dysfunction, asymptomatic atrial fibrillation, and hypertrophic cardiomyopathy. Additionally, these models can accurately predict demographic and phenotypic features, including age, gender, race, and other phenotypes [4–6]. The integration of AI into ECG analysis not only improves diagnostic precision but also enables timely and targeted interventions, ultimately leading to better patient care and improved health outcomes.

Recent advancements in artificial intelligence, particularly deep learning models such as convolutional neural networks (CNNs) and long short-term memory (LSTM) networks [7–9], have revolutionized the field of ECG interpretation [10]. These AI-driven approaches have demonstrated exceptional accuracy in detecting nuanced signals and patterns that are typically undetectable by human experts, elevating the ECG to a highly effective, non-invasive biomarker for cardiovascular diagnostics. Despite these achievements, the classification of arrhythmias remains a formidable challenge due to the diverse and complex etiologies underlying ECG signal abnormalities. These abnormalities can be attributed to a multitude of factors, including coronary heart disease, heart failure, cardiac pacing, hypertension, cardiomyopathy, heart valve disease, and electrolyte imbalances. The heterogeneity of these contributing factors significantly complicates the task of accurately classifying arrhythmias. Furthermore, the combination of variational mode decomposition (VMD) and machine learning is an emerging and promising approach for analyzing and modeling complex signals, particularly in time-series analysis and predictive modeling. [11] VMD is a signal decomposition technique that decomposes a signal into intrinsic mode functions (IMFs), while machine learning excels at learning complex patterns from data. Integrating VMD and machine learning can leverage the strengths of both methods to improve model performance and interpretability.

The majority of existing deep-learning techniques for arrhythmia classification are primarily based on ECG data, often neglecting the fundamental principles of cardiac electrophysiology [3]. This limitation underscores the need for a more integrated approach that combines the strengths of AI with a solid understanding of the underlying physiological mechanisms. In this context, this paper seeks to bridge this gap by incorporating the mathematical foundations of cardiac electrophysiology into AI-driven ECG analysis, thereby enhancing the accuracy and interpretability of arrhythmia classification.

This paper presents a framework for modeling mechanisms in arrhythmia signal classification, organized as follows. In Theoretical foundations for electrocardio signal modeling and processing section, the theoretical foundations for electrocardio signal modeling and processing were elaborated, including Hodgkin-Huxley model, finite element method and variational mode decomposition. The finite element model and various arrhythmia models induced by abnormalities of PDEs were established in Modeling of the human heart and arrhythmia electrocardiosignal acquisition section, and the typical synthetic signals of the models were prepared. In Variational Mode Decomposition based on Multi-objective COA section, the multi-objective crayfish optimization algorithmic variational mode decomposition was proposed and the optimal IMFs were obtained. The deep attention model for classification was constructed in Learning model establishment and optimization for classification section, the modules were validated by ablation studies and the hyperparameters were optimized by the Bayesian algorithm. Finally, the Conclusion section offered the discussions and concluding remarks.

## Related works

As noted in the preceding section, a number of AI-based ECG classification methods have been presented in the literature.

Shu Lih Oh *et al*. [7] propose an automated system using a combination of CNN and LSTM for the diagnosis of normal sinus rhythm, left bundle branch block (LBBB), right bundle branch block (RBBB), atrial premature beats (APB) and premature ventricular contraction (PVC) on ECG signals. The model achieves an accuracy of 98.10%, sensitivity of 97.50% and specificity of 98.70% using a ten-fold cross validation strategy. A similar architecture of the deep model consisting of CNN and LSTM can be seen in [8], which achieved an average accuracy of 97.15%.

Sumanta Kuila *et al*. [12, 13] utilize leading algorithms such as K-nearest neighbor, artificial neural network, and support vector machine to classify different features of ECG signals. In their work, a novel classification algorithm is proposed based on ELM (Extreme Learning Machine) with Recurrent Neural Network (RNN) by using morphological filtering. The experimental results with the MIT-BIHdatabase, using hidden neurons of ELM with RNN, show an accuracy of 96.41%, sensitivity of 93.62% and specificity of 92.66%.

Sayli Siddhasanjay Aphale *et al*. [14] proposed a novel convolutional neural network named ArrhyNet for MIT-BIH arrhythmia classification. Low pass filter and baseline wander filter, along with the Synthetic Minority Over Sampling (SMOTE) technique are utilized to enhance the performance. The results indicate that the top-1 accuracy of the fiveclass classification system for the database used is 92.73%.

Sadegh Ilbeigipour *et al*. [15] developed and compared three decision trees, random forest, and logistic regression classifiers, respectively. The performance of the random forest classifier was much higher than that of the decision trees and logistic regression. The model was tested on the MIT-BIH database and achieved 88% accuracy for 3-class classifications.

Qi Meng *et al*. [16] propose a multi-database integration methodology and a heartbeat self-processing method to eliminate data differences. A 16-classification 5-layer fully connected neural network was built and verified based on the integrated three databases Hercules-3, achieving an accuracy rate of up to 98.67%. Hany El-Ghaish *et al*. [17] develop a deep learning framework called ECGTransForm by embedding a novel Bidirectional Transformer (BiTrans) mechanism, ensuring a robust spatial feature extraction across various granularities.

Currently, most AI-driven models for arrhythmia classification focus primarily on ECG data, ignoring the fundamentals of cardiac electrophysiology. As a result, this limitation highlights the need for a more integrated approach that combines AI’s strengths with a solid understanding of physiological mechanisms. A precise mathematical model of the ECG needs to be developed, and the accuracy of predictions made by deep models will also significantly improve. Using the mathematical foundations of cardiac electrophysiology, this paper aims to bridge the gap between electrophysiology and AI-driven ECG analysis.

## Theoretical foundations for electrocardio signal modeling and processing

### Modelling theory of cardiac electrophysiology

Biological systems exhibit numerous electrical activities in response to external stimuli, which are crucial for neuron-based application in information processing analysis. However, the impact of these electrical activities on the encoding and decoding technologies in neuroscience has not yet been fully understood or well established.

There are various neuron models developed to imitate certain biological neuron dynamics [18–21], The Hodgkin-Huxley (HH) model is a mathematical model that describes how action potentials in neurons are initiated and propagated. This model was developed by Alan Hodgkin and Andrew Huxley in 1952 based on their experimental work on the giant axon of the squid [22]. The HH model is considered one of the most influential models in neuroscience due to its accuracy in describing the electrical properties of neurons and its ability to explain the underlying mechanisms of neuronal excitability. Subsequently, variants based on the HH model emerged, such as the FitzHugh-Nagumo (FHN) model [18, 23], a simplified version of the Hodgkin-Huxley model, which captures the essential features of excitability and action potential generation in neurons [24]. The FHN model reduces the complexity of the full Hodgkin-Huxley model while preserving the key dynamics of spike generation and propagation, which facilitates the analysis of fundamental action potential and excitability dynamics, broadening its applicability for studying excitable systems in a more accessible mathematical framework.

The Hodgkin-Huxley model is described by a set of four coupled nonlinear ordinary differential equations. The membrane potential equation is expressed by

$$C_{m}\frac{dV}{dt}=I-{\bar{g}}_{\text{Na}}m^{3}\;h(V-E_{\text{Na}})-{\bar{g}}_{K}n^{4}(V-E_{K})-{\bar{g}}_{L}(V-E_{L})$$ (1)

Where *V* is the membrane potential, *I* is the external applied current, *C*_*m*_ is the membrane capacitance, ${\bar{g}}_{\text{Na}}$, ${\bar{g}}_{K}$, ${\bar{g}}_{L}$ are the maximum conductances for sodium (${\text{Na}}^{+}$), potassium (${\text{K}}^{+}$), and leak currents, respectively. $E_{\text{Na}}$, *E*_*K*_, *E*_*L*_ are the reversal potentials for sodium, potassium, and leak currents, respectively.

*m*, *h*, and *n* are gating variables representing the sodium activation, sodium inactivation and potassium activation channels, respectively, whose dynamics can be modeled by gating variable equations


$$\frac{dm}{dt}=\alpha_{m}(V)(1-m)-\beta_{m}(V)m$$

$$\frac{dh}{dt}=\alpha_{h}(V)(1-h)-\beta_{h}(V)h$$ (2)

$$\frac{dn}{dt}=\alpha_{n}(V)(1-n)-\beta_{n}(V)n$$

where α and β are rate functions that describe the opening and closing rates of the ion channels, which are defined by


$$\alpha_{m}(V)=\frac{0.1(V+40)}{1-e^{-(V+40)/10}}$$

$$\beta_{m}(V)=4e^{-(V+65)/18}$$

$$\alpha_{h}(V)=0.07e^{-(V+65)/20}$$ (3)

$$\beta_{h}(V)=\frac{1}{1+e^{-(V+35)/10}}$$

$$\alpha_{n}(V)=\frac{0.01(V+55)}{1-e^{-(V+55)/10}}$$

$$\beta_{n}(V)=0.125e^{-(V+65)/80}$$

### Finite element method for numeric solving

Developed in the early 1960s, the finite element method (FEM) has become one of the most widely used numerical analysis approaches in engineering. By simulating the physical behavior of objects and systems, engineers are able to optimize designs, ensure safety, and reduce development costs [25]. Consider a multidimensional steady-state heat conduction process as an example, which can be described by the Poisson equation with homogeneous boundary conditions

$$\begin{array}{c} -\nabla^{2}u=f\;\text{in }\Omega ,\\ u=0\;\text{on}\;\partial \Omega , \end{array}$$ (4)

with domain $\Omega \subset {\mathbb{R}}^{d}$. Here, *u* is the unknown function to be solved, and *f* is the known function (source term). The weak form of Eq 4 can be obtained by choosing a function *v* from a space *U* of smooth functions by performing the inner product of both sides with *v*, i.e.,

$$-\left\langle \nabla^{2}u,v\right\rangle =\langle f,v\rangle$$ (5)

Assume that, in addition to having the necessary smoothness, the functions that are to be the solutions also satisfy the boundary conditions. The space *U* of test functions is of the form $U=\{v:v\in C^{2}(\Omega ),v=0$ on ∂Ω}. More specifically, let d = 2, the weak form of Eq 4 can be written as

$$\iint_{\Omega }\nabla u\cdot \nabla vdxdy=\iint_{\Omega }fvdxdy.$$ (6)

To obtain a numerical method, it requires *U* to be finite-dimensional with basis $\left\{u_{1},\ldots ,u_{n}\right\}$. Then the approximate solution ${\text{u}}^{\text{h}}$ of Eq 4 can be represented as

$$u^{h}=\sum_{j=1}^{n}c_{j}u_{j}.$$ (7)

where the coefficients *c*_*j*_ are to be determined once a basis has been chosen for the approximation space *U*. By inserting ${\text{u}}^{\text{h}}$ into the weak form Eq 6, and selecting the basis functions of *U* as trial functions *v*, a system of equations is obtained

$$\iint_{\Omega }\nabla \left[\sum_{j=1}^{n}c_{j}u_{j}\right]\cdot \nabla u_{i}dxdy=\iint_{\Omega }fu_{i}dxdy,\;i=1,\ldots ,n$$ (8)

Which is known as the Ritz-Galerkin method and can be written in matrix form, A𝐜=𝐛, The expression of stiffness matrix *A* is

$$A_{i,j}=\iint_{\Omega }\nabla u_{j}\cdot \nabla u_{i}dxdy$$ (9)

### Theoretical basis of variational mode decomposition

Variational Mode Decomposition (VMD) [26] represents a recent advancement in signal processing, designed to decompose a multi-component signal *f* into a series of *K* quasi-orthogonal intrinsic mode functions (IMFs) *u*_*k*_. Each IMF exhibits specific sparsity characteristics and limited bandwidth, obtained through a non-recursive decomposition process. The variational formulation and the optimization-based approach make VMD a powerful and flexible tool for multi-component signal analysis and decomposition, with advantages over traditional data-driven methods like Empirical Mode Decomposition (EMD) [27], Ensemble Empirical Mode Decomposition (EEMD) [28], etc., which recursively decomposes a non-stationary signal into IMFs thus facing robustness and mode mixing problems [29].

The essential aspect of the VMD is solving a constrained variational formulation written as

$$\min_{\left\{u_{k}\right\},\left\{\omega_{k}\right\}}\left\{\sum_{k}{\left\|\partial_{t}\left[\left(\delta (t)+\frac{j}{\pi t}\right)\times u_{k}(t)\right]e^{-j\omega_{k}t}\right\|}_{2}^{2}\right\},\;\text{s.t.}\;\sum_{k}u_{k}=f$$ (10)

Where $\left\{u_{k}\right\}:=\left\{u_{1},\ldots ,u_{K}\right\}$ and $\left\{\omega_{k}\right\}:=\left\{\omega_{1},\ldots ,\omega_{K}\right\}$ are shortening designations for the set of all modes and their center frequencies, respectively. $\partial_{t}$ is the partial derivative of the function at the time *t*, and δ(t) is the unit impulse function. $\sum_{k}:=\sum_{k=1}^{K}$ denotes the summation over all modes. In order to render the problem unconstrained, both a quadratic penalty term α and a Lagrangian multiplier λ are considered, and the augmented Lagrangian ℒ is introduced as

$$\begin{array}{ccc} ℒ\left(\left\{u_{k}\right\},\left\{\omega_{k}\right\},\lambda \right) & := & \alpha \sum_{k}{\left\|\partial_{t}\left[\left(\delta (t)+\frac{j}{\pi t}\right)*u_{k}(t)\right]e^{-j\omega_{k}t}\right\|}_{2}^{2}\\ & & +{\left\|f(t)-\sum_{k}u_{k}(t)\right\|}_{2}^{2}+\left\langle \lambda (t),f(t)-\sum_{k}u_{k}(t)\right\rangle \end{array}$$ (11)

Therefore, the original minimization problem Eq 10 can be solved by finding the saddle point of the augmented Lagrangian in a sequence of iterative sub-optimizations known as the alternating direction method of multipliers (ADMM) [30]. After pre-setting the decomposition mode number *K* and the quadratic penalty term α, the decomposed mode ${\hat{u}}_{k}$ and its associated center frequency $\omega_{k}^{n+1}$, along with the Lagrangian multiplier $\hat{\lambda }$, are initialized. Then the updating strategy of modes *u*_*k*_(*t*) for the *k*-th mode is

$${\hat{u}}_{k}^{n+1}\left(\omega \right)\leftarrow \frac{\hat{f}\left(\omega \right)-\underset{i<k}{\sum }{\hat{u}}_{i}^{n+1}\left(\omega \right)-\underset{i>k}{\sum }{\hat{u}}_{i}^{n}\left(\omega \right)+\frac{{\hat{\lambda }}^{n}\left(\omega \right)}{2}}{1+2\alpha {\left(\omega -\omega_{k}^{n}\right)}^{2}}$$ (12)

where the ${\hat{u}}_{k}\left(\omega \right)$ is the Fourier transform of the *k*-th mode, $\hat{f}\left(\omega \right)$ is the Fourier transform of the original signal, $\hat{\lambda }(\omega )$ is the Fourier transform of the Lagrangian multiplier, and *n* is the iteration index. The center frequencies $\omega_{k}$ are updated to minimize the bandwidth of each mode by

$$\omega_{k}^{n+1}\leftarrow \frac{\int_{0}^{\infty }\omega {\left|{\hat{u}}_{k}^{n+1}\left(\omega \right)\right|}^{2}d\omega }{\int_{0}^{\infty }{\left|{\hat{u}}_{k}^{n+1}\left(\omega \right)\right|}^{2}d\omega }$$ (13)

The Lagrangian multiplier λ(t) is updated to enforce the constraint that the sum of the modes equals the original signal by

$${\hat{\lambda }}^{n+1}\left(\omega \right)\leftarrow {\hat{\lambda }}^{n}\left(\omega \right)+\tau \left(\hat{f}\left(\omega \right)-\underset{k}{\sum }{\hat{u}}_{k}^{n+1}\left(\omega \right)\right)$$ (14)

where τ is the update parameter for the Lagrangian multiplier. The criterion of convergence is

$$\sum_{k}\frac{{\left\|\begin{array}{c} {\hat{u}}_{k}^{n+1}-{\hat{u}}_{k}^{n} \end{array}\right\|}_{2}^{2}}{{\left\|\begin{array}{c} {\hat{u}}_{k}^{n} \end{array}\right\|}_{2}^{2}}<\varepsilon$$ (15)

where ε is typically set to 10^−7^, the value of *k* is constrained by the complexity of the signal and computational resources, normally less than 10. Finally, the K modes of the original signal are obtained.

The selection of the number of intrinsic mode functions *K* and the penalty term α is paramount when implementing the variational mode decomposition method. These parameters significantly influence the decomposition process and the quality of the resulting intrinsic mode functions. *K* influences the granularity of the decomposition, while α controls the smoothness of the resulting modes. Therefore, optimizing the number of intrinsic mode functions and the penalty term in Variational Mode Decomposition is crucial for achieving accurate and meaningful signal decomposition, which balances the need to capture all relevant signal components against the risk of introducing noise or artifacts. Various optimization methods, such as grid search, Bayesian optimization, and meta-heuristic optimization can be used to achieve the best decomposition results [31–33].

Popular meta-heuristic optimization techniques are typically inspired by physical phenomena, species’ behaviors, biological evolution, or laws governing social operations [34, 35]. Many emergent behavioral patterns in organisms have optimized overtones due to the evolutionary process of natural selection and have been identified separately and used in nature-inspired algorithms. There are plenteous evolutionary algorithms and swarm algorithms built on the foundations of naturally occurring mechanisms, such as Genetic Algorithm (GA) [36], Particle Swarm Optimization (PSO) [37], Gray Wolf Optimization (GWO) [38], Gold Rush Optimizer (GRO) [39], Rime Optimization Algorithm (RIME) [40], Crayfish optimization algorithm (COA) [41], Beluga Whale Optimization (BWO) [42] and so on. Meta-heuristic optimization algorithms are playing an increasingly important role in optimization and decision-making problems in various engineering fields and will also be employed in this paper.

### Attention scheme for sequencing signal feature extraction

The Transformer model [43] has proven a great success in sequencing signal feature extraction, making a significant contribution to natural language processing (NLP) tasks, and has become the foundation for many state-of-the-art deep network models, such as BERT [44], GPT [45] and so on. Effectively capturing long-range dependencies and relationships in the input sequence is made possible by the attention scheme, which is a key component and a powerful mechanism of the Transformer model. Instead of processing the entire input sequence uniformly, the attention mechanism enables the model to selectively focus on relevant parts of the input when generating the output.

The attention scheme in the Transformer model mainly consists of three matrix components, i.e., Query (*Q*), Key (*K*), and Value (*V*), which is linearly transformed by the input sequences. The dot product between *Q* and *K* is calculated and is divided by $\sqrt{d_{k}}$ to prevent the result from being too large, where *d*_*k*_ is the dimension of the Key vector. After implementing the Softmax operation to normalize the results, the probability distribution is obtained, which is finally multiplied by the matrix *V* to yield the weighted sum representation as the final output, as shown in Eq 16.

$$\mathrm{Attention}(Q,K,V)=\mathrm{softmax}\left(\frac{QK^{T}}{\sqrt{d_{k}}}\right)V$$ (16)

The Transformer model utilizes multiple attention heads, each with its own set of Query, Key, and Value matrices, which facilitate the model to capture different types of relationships and dependencies in the input sequence.

$$\begin{array}{ccc} \mathrm{MultiHead}\left(Q,K,V\right) & = & \mathrm{Concat}\left({\mathrm{head}}_{1},\ldots ,{\mathrm{head}}_{h}\right)W^{O}\\ \text{where}\;{\text{head}}_{i} & = & \mathrm{Attention}(W_{i}^{Q},KW_{i}^{K},VW_{i}^{V}) \end{array}$$ (17)

where the projections are parameter matrices $W_{i}^{Q}\in {\mathbb{R}}^{d_{\text{model}}\times d_{k}},W_{i}^{K}\in {\mathbb{R}}^{d_{\text{model }}\times d_{k}},W_{i}^{V}\in {\mathbb{R}}^{d_{\text{model }}\times d}$ and $W^{O}\in {\mathbb{R}}^{hd_{v}\times d_{\text{model }}}$ .

The position-wise feed-forward network performs two linear transformations with a ReLU activation function [46] that can be modeled as

$$\mathrm{FFN}\left(x\right)=\mathrm{ReLU}(xW_{1}+b_{1})W_{2}+b_{2}$$ (18)

where *W*_1_, *W*_2_ are the weights and *b*_2_ is the bias of linear transformations, which is the same across different positions but vary from layer to layer. Each layer of the Transformer Encoder module consists of two primary components, multi-head self-attention mechanisms and position-wise feed-forward networks. All the parts work together to interpret the input sequence into a high-level abstraction, prospering the transformer model a powerful architecture for dealing with sequential data.

## Modeling of the human heart and arrhythmia electrocardiosignal acquisition

### Finite element model establishment

**Fig 1:**
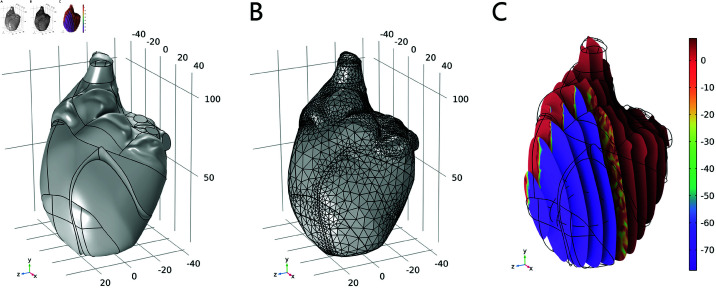
Model establishment of the human heart. (A) 3D geometry. (B) Finite element model. (C) Initial condition.

The task of modeling the three-dimensional human heart is intricate and requires the integration of data from multiple imaging modalities, anatomical knowledge, and computational techniques. In specific, firstly, acquire high-resolution imaging from Magnetic Resonance Imaging (MRI) data, then improve image quality by applying cleaning and preprocessing algorithms to prepare the data for segmentation, and extract the heart structures from the imaging data. Finally, a 3D model is converted by the segmented 2D slices by stacking and interpolating between the slices, which can be rendered by visualization tools. In this paper, a full-scale, three-dimensional model of the human heart is established by MRI data, as shown in Fig 1(A).

The common values of the parameters in HH model are examined and determined by the following. $C_{m}\approx 1\;\mu {\text{F/cm}}^{2}$, ${\bar{g}}_{\text{Na}}\approx 120\;{\text{mS/cm}}^{2}$, ${\bar{g}}_{K}\approx 36\;{\text{mS/cm}}^{2}$, ${\bar{g}}_{L}\approx 0.3\;{\text{mS/cm}}^{2}$, $E_{\text{Na}}\approx 50\;\text{mV}$, $E_{K}\approx -77\;\text{mV}$, $E_{L}\approx -54.4\;\text{mV}$.

For simulations, the typical initial condition of the membrane potential $V_{0}$ is set to −65mV, and the initial gating variables are usually set to their steady-state values at the resting potential $V_{0}$ calculated by


$$m_{0}=\frac{\alpha_{m}(V_{0})}{\alpha_{m}(V_{0})+\beta_{m}(V_{0})}$$

$$h_{0}=\frac{\alpha_{h}(V_{0})}{\alpha_{h}(V_{0})+\beta_{h}(V_{0})}$$ (19)

$$n_{0}=\frac{\alpha_{n}(V_{0})}{\alpha_{n}(V_{0})+\beta_{n}(V_{0})}$$

The potential distribution *V* is initialized by setting one quadrant of the heart at a constant, elevated potential,$V_{0}$, while the rest remains at zero. The logical expression of the boundary condition can be written as

$$V(0,x,y,z)=V_{0}((x+d)>0)\cdot ((z+d)>0)$$ (20)

Where TRUE evaluates to 1 and FALSE to 0. *d* is set to 10^−5^, which is included in the expressions to shift the elevated potential slightly off the main axes.

The simulation platform *COMSOL Multiphysics* is adopted for partial differential equation (PDE) solving and result visualization, which allows for coupling various physical fields, including cardiac electrophysiology and mechanics. Due to a plethora of irregular and complex surfaces in the 3D model, the free tetrahedron mesh is employed as the partitioning strategy. The finite element mesh model that consists of 30245 elements across all domains is shown in Fig 1(B), and the initial condition of Eq 19 is visualized in Fig 1(C).

**Fig 2:**
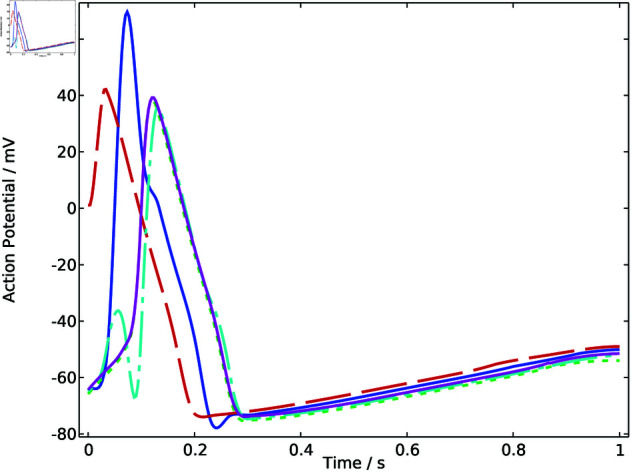
Action potential of sample points in model 0.

Through FEM simulation results, the temporal variation pattern of the action potential throughout the heart model region is revealed. Five representative points within the heart region are extracted for further analysis. As shown in Fig 2, the action potential signals at these representative points exhibit typical and homogeneous electrocardiographic characteristics. The different phases demonstrate the propagation of electrical signals through the heart tissues. For convenience, the original model is named model 0, and the electrocardiographic signals are identified as signal 0.

### Various arrhythmias modelings induced by PDE parameters

According to the Hodgkin-Huxley model, it can be deduced that the parameters in partial differential Eq 1 and gating variable Eq 2 play essential roles in the propagation behaviors of electrocardiographic signals. Therefore, the key anomalies characterized by *I* and *h* in the mathematical model of HH are examined in detail. An exponentially attenuated sinusoidal function is well-designed for simulating the HH model’s abnormal dynamics caused by PDE variables, which is defined as

$$\Psi (t)=e^{-4t}*sin(2\pi t)$$ (21)

**Fig 3:**
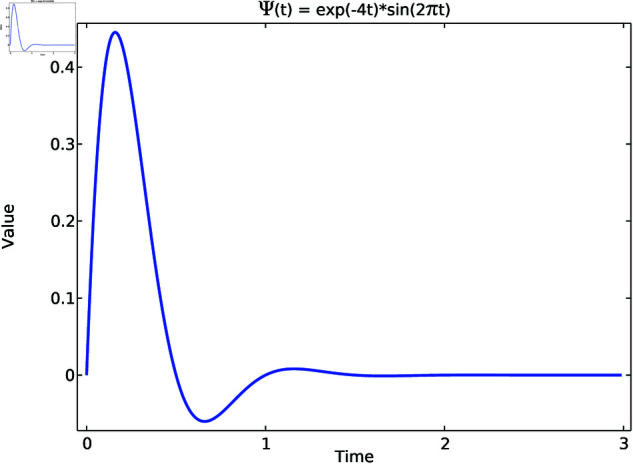
Exponentially attenuated sinusoidal function.

The function consists of two main components: an exponentially decaying term and a sinusoidal term with a frequency of 2π. Fig 3 depicts the varying pattern of the function value over time, which exhibits a sinusoidal oscillation whose amplitude decays exponentially.

The variable *I* is a critical parameter representing the external current applied to the neuron. The underlying dynamics of action potential generation and propagation in cardiac cells can be understood by adjusting this parameter to simulate and study different excitability conditions. Here, consider multiplying *I* by the exponentially attenuated sinusoidal function Ψ(t), then the HH model given by Eq 1 transforms to

$$C_{m}\frac{dV}{dt}=I*\Psi (t)-{\bar{g}}_{\text{Na}}m^{3}\;h(V-E_{\text{Na}})-{\bar{g}}_{K}n^{4}(V-E_{K})-{\bar{g}}_{L}(V-E_{L})$$ (22)

**Fig 4:**
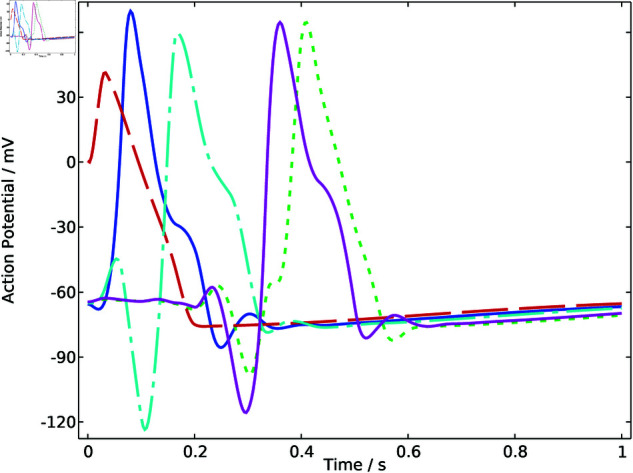
Action potential of sample points in model 1.

The arrhythmia model described by Eq 22 is denoted as model 1. After mesh generation and FEM calculation, the results of five representative points within the heart region are displayed in Fig 4, which are referred to as signal 1. The curves of action potential changing with time show a homogeneous pattern with different amplitudes and phases, which exhibit more fluctuated characteristics compared with signal 0.

The sodium inactivation gate *h* plays a critical role in regulating the availability of sodium channels during and after an action potential. By inactivating the channels during depolarization and allowing them to recover during repolarization, it ensures that action potentials are well-spaced and that the neuron can return to its resting state properly. The exponentially attenuated sinusoidal function Ψ(t) is encapsulated in Eq 2 of the sodium inactivation variable *h*, which is regarded as the arrhythmia model 2 and can be represented by

$$\frac{dh}{dt}=(\alpha_{h}(V)(1-h)-\beta_{h}(V)h)*\Psi (t)$$ (23)

**Fig 5:**
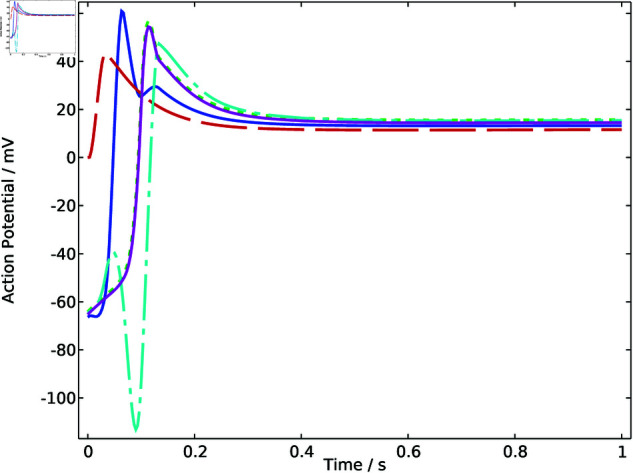
Action potential of sample points in model 2.

Similarly, the arrhythmia model 2 represented by Eq 23 is modeled and calculated using the finite element method. The results for five representative points within the heart region are illustrated in Fig 5. The curves of action potential changing over time demonstrate a homogeneous pattern but show different characteristics compared with signal 0 and signal 1 in terms of amplitude.

**Fig 6:**
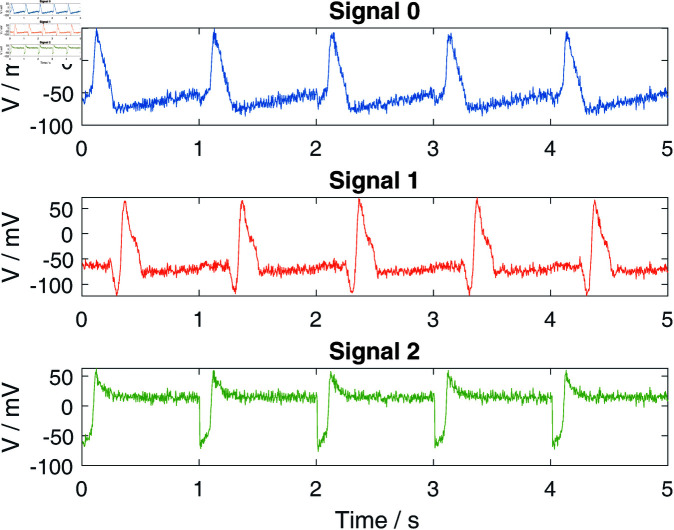
Different patterns of electrocardio signals with Gaussian noise. X-axis represents time, Y-axis represents the magnitude of the membrane potential.

In order to construct the final synthetic signals, the prototypical signals derived from the three models are collected and processed through periodization. Moreover, Gaussian white noise, with a range of signal-to-noise ratios (SNR), is introduced to the synthetic signals to enhance their realism and robustness in various analytical contexts. Fig 6 exhibits the comparison of the typical synthetic signals of the three models with a noise standard deviation of 5 and a mean of -65, which are prepared for the next signal decomposition and classification.

## Variational mode decomposition based on multi-objective COA

### Crayfish optimization algorithm and comparison with competitive meta-heuristic algorithms

The Crayfish Optimization Algorithm (COA) represents a novel meta-heuristic optimization method inspired by the foraging behaviors and navigational strategies observed in crayfish [41]. Similar to other nature-inspired algorithms (such as Genetic Algorithms, Particle Swarm Optimization, and Ant Colony Optimization), COA aims to solve complex optimization problems by mimicking the natural processes observed in crayfish. The feeding amount of crayfish is influenced by temperature, with the ideal feeding range for crayfish being between 20°*C* and 30°*C*, and 25°*C* being the best temperature. Thus, COA defines temperature range from 20 to 35 °*C*

temp=rand×15+20 (24)

where *temp* denotes the ambient temperature of the crayfish’s location, and *rand* is the random scalar obtained from the uniform distribution of the interval (0,1). The mathematical model for crayfish intake is characterized by a Gaussian-like distribution

$$p=C_{1}\times \left(\frac{1}{\sqrt{2\pi }\times \sigma }\times \exp\left(-\frac{{\left(temp-\mu \right)}^{2}}{2\sigma^{2}}\right)\right)$$ (25)

where μ denotes the temperature most suitable for crayfish, σ and *C*_1_ are parameters controlling the intake of crayfish at different temperatures.

The goal of the crayfish at the summer resort stage is to get to the cave, which stands for the ideal solution. By doing so, individuals get closer to the ideal solution and improves COA’s exploitation potential. The crayfish will enter the cave for summer resort by

$$X_{i,j}^{t+1}=X_{i,j}^{t}+C_{2}\times rand\times \left(X_{shade}-X_{i,j}^{t}\right)$$ (26)

where *t*, *t*  +  1 represent the current generation number and the next generation iteration number, respectively. *C*_2_ is a decreasing coefficient. The cave $X_{shade}:=\left(X_{G}+X_{L}\right)/2$, where *X*_*G*_ denotes the optimal position obtained so far by the number of iterations, and *X*_*L*_ refers to the optimal position of the current population.

In the Competition stage, crayfish *X*_*i*_ engage in competition with each other and modify their positions in response to the position *X*_*z*_ of another crayfish. Adjusting the position expands the search range of COA. The crayfish compete for the cave through

$$X_{i,j}^{t+1}=X_{i,j}^{t}-X_{z,j}^{t}+X_{shade}$$ (27)

where *z* represents the random individual of crayfish calculated by z=round(rand × (N − 1))  +  1, *N* denoting the population size.

In the Foraging stage, crayfish use different feeding methods based on the size of their food *Q*

$$Q=C_{3}\times rand\times \left({fitness}_{i}/{fitness}_{food}\right)$$ (28)

where *C*_3_ is the food factor, representing the largest food. *fitness*_*i*_ represents the fitness value of the *i*-th crayfish, and *fitness*_*food*_ represents the fitness value of the food location. The crayfish will approach the food *Q* when it is the right size for eating. When *Q* is too large ($Q>\left(C_{3}+1\right)/2$), it indicates that there is a significant difference between the crayfish and the optimal solution. At this time, the crayfish will tear the food with the first claw and the mathematical equation is

$$X_{food}=\exp\left(-\frac{1}{Q}\right)\times X_{food}$$ (29)

The food obtained by crayfish is also related to the food intake, so the equation for foraging is as follows

$$X_{i,j}^{t+1}=X_{i,j}^{t}+X_{food}\times p\times \left(\cos\left(2\times \pi \times rand\right)-\sin\left(2\times \pi \times rand\right)\right)$$ (30)

where food *X*_*food*_ represents the optimal solution, which is supposed to be reduced and brought closer to the food. When the size of the food is not too large ($Q\leq \left(C_{3}+1\right)/2$), the crayfish can simply move towards the food and eat directly

$$X_{i,j}^{t+1}=\left(X_{i,j}^{t}-X_{food}\right)\times p+p\times rand\times X_{i,j}^{t}$$ (31)

Through the foraging stage, COA will approach the optimal solution, improving the algorithm’s exploitation ability and allowing it to have excellent convergence capabilities.

A well-designed test function, named Ackley’s Function, is built and used to verify the performance of COA. Transformations such as shifts and rotations are introduced to increase the complexity of the function landscape. As a result, this function poses significant challenges for global optimization algorithms due to its numerous local minima and a single global minimum. The expression of the test function is

$$f_{\text{Ackley}}(𝐱)=-a\exp\left(-b\sqrt{\frac{1}{d}\sum_{i=1}^{d}x_{i}^{2}}\right)-\exp\left(\frac{1}{d}\sum_{i=1}^{d}\cos(cx_{i})\right)+a+e$$ (32)

where $𝐱=(x_{1},x_{2},\ldots ,x_{d})$ is a vector of variables, *d* is the dimension of the search space. *a*, *b*, and *c* are constants, here *a* = 20, *b* = 0.2, and c=2π. *e* is the base of the natural logarithm.

**Fig 7:**
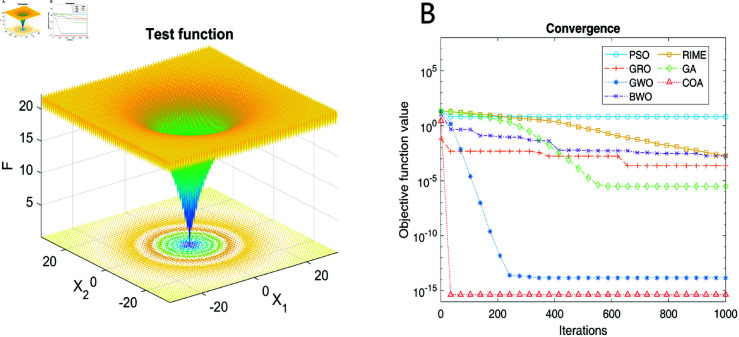
Comparison of different optimization algorithms. (A) Schematic diagram of test function. (B) Convergence comparison.

The function landscape is displayed in Fig 7(A), where the range of **x** is [–32,32] in each dimension, and the colormap corresponds to the function value. Several state-of-the-art algorithms mentioned before, i.e., GWO, GRO, RIME, PSO, GA, and BWO were chosen as competitors, the convergence comparison of which is shown in Fig 7(B). After 1000 iterations, GA has the best precision followed by COA, whereas the other algorithms exhibit premature convergence and remain steady. However, it is evident that COA converges faster than GA, which falls into the local optimum in hundreds of iterations during optimization. In summary, COA shows the greatest potential among other algorithms and is worth further development for multi-objective optimization.

### Multi-objective COA based on non-dominated sorting

The original Crayfish Optimization Algorithm (COA) is formulated for scalar objective optimization, where a single objective function is optimized. However, in many practical engineering applications, multi-objective optimization problems are prevalent. These problems involve the simultaneous optimization of multiple, often conflicting, objectives, which adds a layer of complexity not present in single-objective optimization. For example, solutions to a problem with two objectives are not straightforward, as improving one objective may lead to the deterioration of another. Therefore, it is imperative to enhance the COA to broaden its applicability for multi-objective optimization problems. Regarding this point, a Multi-objective COA (MOCOA) based on non-dominated sorting is proposed in this paper.

Non-dominated sorting is an essential concept in multi-objective optimization, particularly in evolutionary algorithms. It is used to classify solutions based on Pareto dominance, helping to identify the set of optimal solutions known as the Pareto front [47].

For the minimization optimization problem with a feasible region Ω, a vector $U=\left[u_{1},u_{2},\ldots ,u_{k}\right]\in {\mathbb{R}}^{k}$ is dominant to vector $V=\left[v_{1},v_{2},\ldots ,v_{k}\right]\in {\mathbb{R}}^{k}$ (denoted by U≺V) if and only if $\forall i\in \left\{1,2,\ldots ,k\right\},u_{i}\leq v_{i}$ ∧ $\exists j\in \left\{1,2,\ldots ,k\right\}:u_{j}<v_{j}$. That is to say, there is at least one *u*_*j*_ which is smaller than $v_{j}$ whilst the remaining *u*’s are either smaller or equal to corresponding *v*’s. The solution vector ${\text{X}}^{*}$ is considered Pareto optimal (minimal) if no other solution can be found to dominate ${\text{X}}^{*}$ according to the definition of Pareto dominance, i,e., ∀i∈{1,2,…,k},∀X∈Ω − $\left\{X^{*}\right\},f_{i}\left(X^{*}\right)\leq f_{i}\left(X\right)$ ∧ $\exists j\in \{1,2,\ldots ,k\}:f_{j}\left(X^{*}\right)<f_{j}\left(X\right)$. A Pareto set is a set in the decision variable space consisting of all the Pareto optimal vectors $P^{*}=\left\{X\in \Omega |∄X^{\prime }\in \Omega :F\left(X^{\prime }\right)≺F\left(X\right)\right\}$. The Pareto front ${\text{PF}}^{*}$is a set of vectors of objective functions generated from the vectors of decision variables in the Pareto set $PF^{*}=\left\{F\left(X\right)=\left(f_{1}\left(X\right),f_{2}\left(X\right),\ldots ,f_{k}\left(X\right)\right):X\in P^{*}\right\}$.

Non-dominated sorting is a fundamental technique in multi-objective optimization to classify solutions based on Pareto dominance. Solutions are grouped into different fronts based on their dominance relationships, and each front is assigned a rank, which denotes the level of Pareto dominance it belongs to. For example, solutions in the same rank are equally non-dominated with respect to each other. Lower ranks (e.g., Rank 1) indicate solutions that are closer to the Pareto front, representing more optimal trade-offs among the objectives.

The concept of crowding degree is further introduced, which represents the density of the surrounding individuals. As the crowding degree decreases, the density of surrounding individuals increases. Consequently, individuals with greater crowding distance at the same rank are selected. The crowding degree is computed by

$$\text{distance}\left[i\right]=\frac{f\left(x_{j\_i+1}\right)-f\left(x_{j\_i-1}\right)}{f_{j\_\max}-f_{j\_\min}}$$ (33)

where $f\left(x_{j\_i+1}\right)$ and $f\left(x_{j\_i-1}\right)$ refer to the two adjacent fitness values of the *i*-th individual in the *j*-th rank, respectively. $f_{j\_\max}$ is the maximum fitness value, and $f_{j\_\min}$ is the minimum fitness value in the *j*-th rank.

The non-dominated sorting method in this paper can be mainly divided into two steps. The first step is to sort all individuals by using a fast non-dominated sorting algorithm. Then the crowding of the decision space $\left(CD_{i,x}\right)$ and the object space (*CD*_*i*,*f*_) of the *i*-th individual are calculated, respectively. The *i*-th individuals in the same rank are rated based on the special crowding distance

$$SCD_{i}=\left\{ \begin{array}{cc} \max(D_{i,x},CD_{i,f}) & CD_{i,x}>CD_{avg,x}\text{ or }CD_{if}>CD_{avg,f}\\ \min(D_{i,x},CD_{i,f}) & \text{ otherwise } \end{array}\right.$$ (34)

where $CD_{avg,f}$ and $CD_{avg,x}$ denote the average crowding degree of the objective space and the decision space, respectively.

Step 1. Define the number of iterations *T*, population size *N*, dimension of design space *dim*, upper bound *ub*, and lower bound *lb*. For optimization target *F*_*k*_ with *K* different objective functions, initialize population *X*_*i*,*j*_ derived from the upper and lower bounds, respectively

$$P_{F}(X)=\left[\begin{array}{ccccc} X_{1,1} & \cdots & X_{1,j} & \cdots & X_{1,dim}\\ \vdots & \ddots & \vdots & \ddots & \vdots \\ X_{i,1} & \cdots & X_{i,j} & \cdots & X_{i,dim}\\ \vdots & \ddots & \vdots & \ddots & \vdots \\ X_{N,1} & \cdots & X_{N,j} & \cdots & X_{N,dim} \end{array}\right]$$ (35)

*X*_*i*,*j*_ is calculated by $X_{i,j}=lb_{j}+\left(ub_{j}-lb_{j}\right)\times rand$, where *lb*_*j*_, *ub*_*j*_ represent the lower bound and the upper bound of the *j*-th dimension, respectively. Every crayfish is a matrix with dimensions of 1 × *dim*, where each column matrix represents a solution to a problem. Moreover, the ambient temperature of crayfish is determined by Eq 24 to induce different stages of COA.

Step 2. When *temp* > 30 and *rand* < 0.5, COA activates the summer resort stage, a new position $X_{i,j}^{t+1}$ is generated according to the cave position *X*_*shade*_ and crayfish position $X_{i,j}^{t}$ by Eq 26. When *temp* > 30 and rand≥0.5, COA enters the competitive stage, in which two crayfish will compete for the cave, a new position $X_{i,j}^{t+1}$ is obtained based on the cave position (*X*_*shade*_) and the positions ($X_{i,j}^{t}$, $X_{z,j}^{t}$) of the two crayfish by Eq 27.

Step 3. When temp≤30, COA proceeds to the foraging stage, the food intake *p* and the food size *Q* are specified by Eq 25 and 28, respectively. If $Q>\left(C_{3}+1\right)/2$, shred the food according to Eq 29, then update the position through Eq 30. Otherwise, the position of crayfish is updated by Eq 31.

Step 4: Evaluate the fitness of $X_{1},\cdots X_{N}$ at step *t*  +  1 using the different objective functions, respectively, and merge the populations by non-dominated sorting. Specifically, the population at step *t* + 1 is expanded as $X=\{X_{1}^{F_{1}},\cdots ,X_{N}^{F_{1}}\cdots X_{1}^{F_{k}},\cdots ,X_{N}^{F_{k}}\}$, and then non-dominated sorting is conducted based on optimization fitness values *F*(*X*). The special crowding distance of each individual is further computed as the sort criterion for individuals at the same rank. Eventually, the population consisting of *kN* individuals is fully sorted, and only the top *N* individuals are retained as the updated population for the subsequent step.

Step 5: Determine whether to end the cycle based on whether the maximum number of iterations *T* has been reached. If the maximum number of iterations has not been reached, continue with Step 2. Otherwise, output the Pareto optimal front ${\text{PF}}^{*}$.

COA is remarkable in its simulations of the summer resort mechanism, competition mechanism, and foraging mechanism of crayfish, showing significant advantages over other meta-heuristic algorithms. The non-dominated sorting and crowding degree sorting algorithm is innovatively introduced in the MOCOA proposed in this paper, thereby enhancing its applicability and efficacy in resolving multi-objective optimization problems.

### Optimal VMD based on MOCOA

MOCOA-VMD (optimized VMD based on MOCOA) is proposed to search for the best number of intrinsic mode functions *K* and the penalty term α in Variational Mode Decomposition. In order to evaluate the effectiveness of VMD, the kurtosis and KL divergence are introduced as indicators of performance. The spectral kurtosis (SK) is a dimensionless time series statistic used to identify and quantify non-stationary changes in a signal that may reflect the random distribution of time series data. For a given frequency bin, SK measures the deviation of the power spectral density (PSD) from that expected for a Gaussian random process. A high SK level corresponds to a high level of nonstationary or non-Gaussian behavior [48, 49]. The Kurtogram is a graphical representation that shows the spectral kurtosis values of various frequency bands in a signal, and it is used to identify the frequency range where the signal has the highest kurtosis, which can indicate the presence of faults or abnormalities in the signal [50].

The spectral kurtosis is calculated by

$$\mathrm{SK}\left(f\right)=\frac{\langle {\left|X\left(f\right)\right|}^{4}\rangle }{\langle {\left|X\left(f\right)\right|}^{2}\rangle^{2}}-2$$ (36)

where *X*_*f*_ is the Fourier transform of a signal at frequency *f*, and ⟨·⟩ is the operator that computes the expectation of a series.

**Fig 8:**
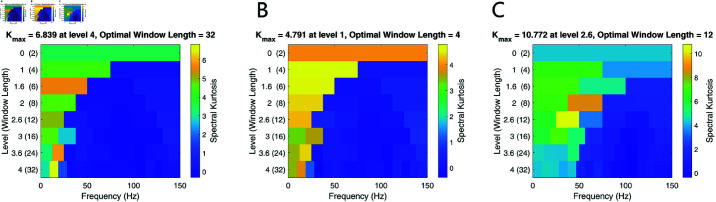
Kurtograms of different signals. (A) Kurtogram of signal 0. (B) Kurtogram of signal 1. (C) Kurtogram of signal 2. X-axis represents the frequency bands, Y-axis represents the resolution levels, color intensity represents the kurtosis value (higher intensity indicates higher kurtosis). Kurtograms exhibit similar impulses components but differentiated characteristics of the kurtosis values across three signals.

Fig 8 shows the Kurtograms of different signals. Among them, the optimal window length of signals are 32, 4,12, respectively, and their kurtograms exhibit similar but differentiated characteristics. The frequency bands with the highest kurtosis values occur at a frequency of around 20 Hz under the sampling frequency of 300 Hz, which indicates the presence of impulses or transients. Kurtosis effectively extracts transient signal changes, making it a suitable performance index.

Kullback–Leibler (KL) divergence quantifies how one probability distribution diverges from another and is a fundamental concept in the realms of information theory and statistics. The discrete form of KL divergence is determined as the index for measuring the similarity between different IMFs, which is defined as

$$D_{KL}(p\|q)=\sum_{i=1}^{N}\left[p\left(x_{i}\right)\log p\left(x_{i}\right)-p\left(x_{i}\right)\log q\left(x_{i}\right)\right]$$ (37)

where *P*(*x*) and *Q*(*x*) are the probabilities of *x* in the distribution of *P* (the true distribution) and *Q* (the approximate distribution), respectively.

**Fig 9:**
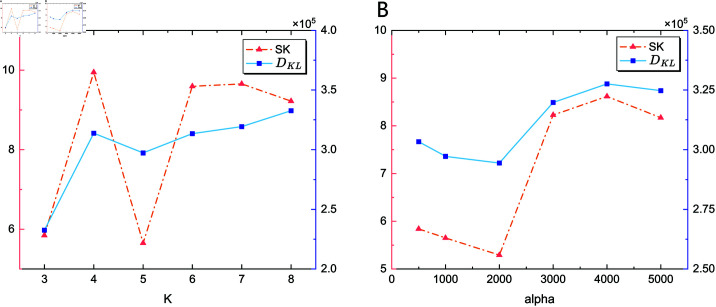
Sensitivity analysis of parameter K and α in VMD. (A) Influence of K on the VMD performance. (B) Influence of α on the VMD performance. X-axis represents the value of *K* or α, left Y-axis represents spectral kurtosis, right Y-axis represents KL divergence. The parameters *K* and α have significant and coupled impacts on decomposition performance.

A sensitivity analysis of *K* and α in VMD was conducted to illustrate how parameter variations affect signal decomposition performance. As shown in Fig 9, the influence of K on the spectral kurtosis and KL divergence is detailed under a fixed value of parameter α=2500; the same applies to α under a fixed *K* = 6. The KL divergence presents a contrary trend as *K* and α rise, and the values of spectral kurtosis fluctuate as *K* and α increase, which reveals the coupling effect of the VMD parameters on the performance indexes.

Sensitivity analysis indicates that *K* and α have varying and coupled impacts on decomposition performance and may interact with each other. Therefore, further optimization is required. The optimization goal for MOCOA is to minimize the spectral kurtosis and KL divergence of the decomposed IMFs by VMD, with 2 design variables *K* and α, to be optimized. Their ranges of design space are integer K∈[3,8] and real number α∈[500,5000], according to previous knowledge. The optimization problem can be written as


$$\arg\min_{x}\;O(x)=[SK_{f}(x),D_{KL}(x){]}^{T}$$

$$\text{s.t.}\;x_{1}=K\in [3,8]\cap \mathbb{Z},$$ (38)

$$x_{2}=\alpha \in [500,5000]\cap \mathbb{R}$$

**Fig 10:**
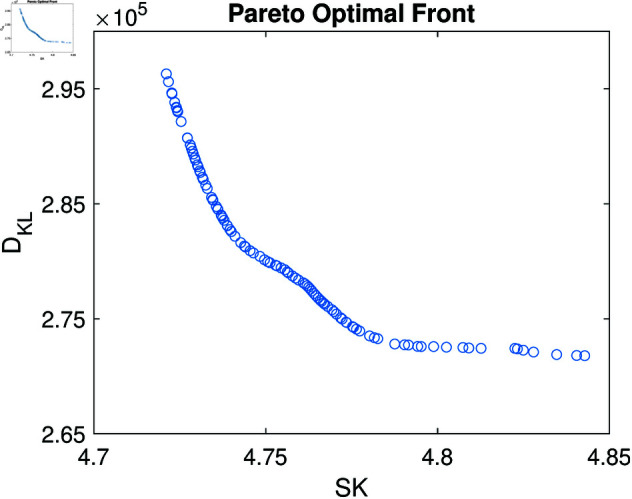
Pareto optimal front result of MOCOA.

Specifically, the MOCOA configuration starts with an initial population of 300 based on the design space, and the maximum number of generations is set to 1000. The spectral kurtosis and KL divergence of each IMFs decomposed by VMD are calculated to evaluate the individual’s performance at each generation continuously. The crowding degree sorting algorithm is employed to rank the individuals in each generation, and the best individuals are selected based on the Pareto dominance relationship. The optimal solution is determined by the Pareto optimal front, which contains the best trade-off solutions between the two objectives. Fig 10 displays the Pareto optimal front after 1000 generations of evolution in the case of signal 0.

Since all the optimal points in the Pareto optimal front’s solution set are mutually non-dominant, any point in the front set could be the optimal candidate. The summary table of the key parameters and performance indices of the VMD is provided in Table 1. The optimal parameters *K* = 5 and α=1928 are determined after subtly selecting, which minimize the spectral kurtosis and KL divergence of the decomposed IMFs. The optimal VMD results are then fed into the deep attention model for arrhythmia classification.

**Table 1:** Summary of key parameters and performance indices of VMD.

Design Params/Metrics	Name	Meaning	Optimal value
Design variable	*K*	number of modes	5
	α	bandwidth control parameter	1928
Performance index	SK	spectral kurtosis	4.74
	*D*_*KL*_	KL divergence	$2.82\times 10^{5}$

**Fig 11:**
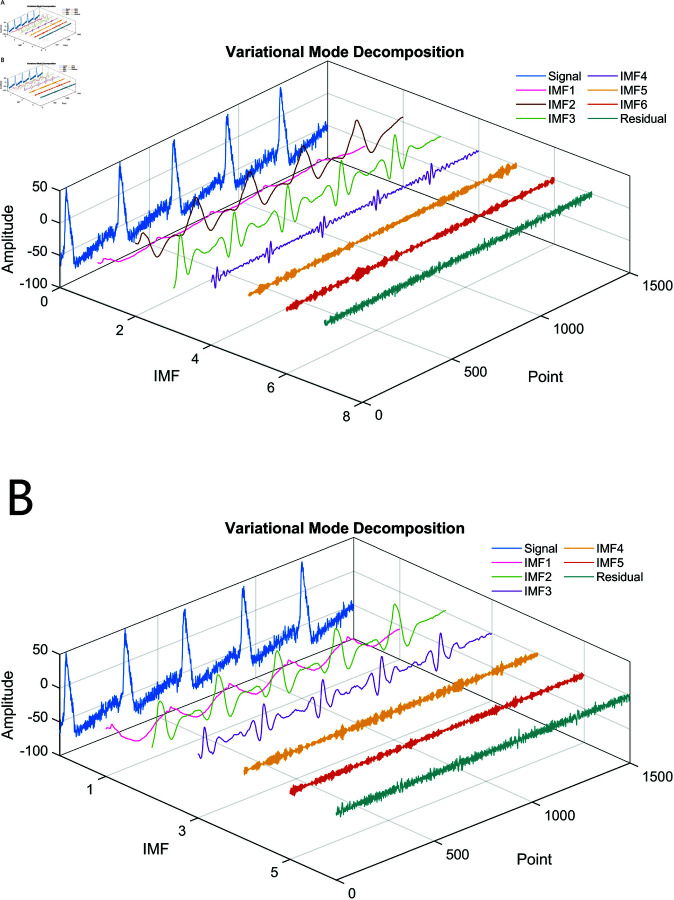
Comparison of original VMD and MOCOA-VMD results. (A) Original VMD result. (B) Optimal VMD result based on MOCOA. IMF1’s MOCOA-VMD result demonstrates superior performance in capturing the main component of the signal compared to the original VMD.

Fig 11 shows the decomposition results of MOCOA-VMD compared with the original VMD with parameters *K* = 6, α=2500. It can be observed that the IMFs decomposed by MOCOA-VMD offers a balance between computational efficiency, decomposition stability, and interpretability, especially in IMF1, where the main component or trend of the signal is detected. The outcome validates the effectiveness of the proposed MOCOA-VMD method, which strikes a compromise between the necessity to capture all pertinent signal components and the potential for the introduction of noise or artifacts.

## Learning model establishment and optimization for classification

### Model establishment based on MOCOA-VMD attention scheme and ablation study

**Fig 12:**
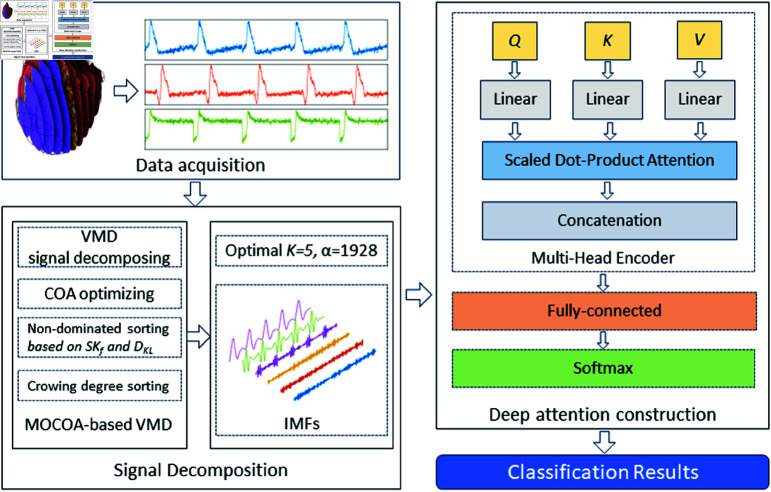
Deep attention network architecture for arrhythmia classification.

The classification of arrhythmias from electrocardiogram signals presents several significant challenges. These challenges often arise from the complex and often non-stationary nature of ECG signals, the substantial variability between patients, and the presence of noise and artifacts that can obscure relevant features. Facing these difficulties, a deep attention model based on MOCOA-VMD is established for arrhythmia signal classification, whose architecture is shown in Fig 12.

During the data acquisition stage, the Hodgkin-Huxley model is simulated and solved using FEM to obtain the ECG signals based on the simulation results. Two different types of arrhythmia signals are artificially generated by introducing the abnormalities in the PDEs. In each case, 200 representative points are uniformly and randomly selected throughout the heart region. The signals from these points are coupled with Gaussian noise at different SNRs. As a result, the total data consists of 3000 signals with 3 different ECG patterns, which are divided into 90% for training and 10% for testing. In the process of signal decomposition, the signals are processed using the proposed MOCOA-VMD method, which employs non-dominated sorting by calculating *K*_*f*_ and *D*_*KL*_ of IMFs and crowding degree sorting. The best decomposition results with the optimal parameters *K* and α in VMD are achieved and the IMF signals are fed to the deep attention model. In deep attention construction, the multi-head encoder consists of matrices *Q*, *K*, and *V* computed by decomposed signals, and the matrices are handled by linear transformation, scaled dot-product attention, and concatenation operation. Then the fully connected layer is employed to fit the features and corresponding categories, and ultimately the softmax layer outputs classification results in the form of a probability distribution.

In order to verify the effectiveness of the proposed signal decomposition method and deep attention modules, an ablation study is implemented, which is a critical part of evaluating the performance and importance of different components in a deep learning model or system. Specifically, the various components in the model are systematically removed or altered to understand their contribution to the overall performance and which parts might be redundant or less important.

The ablation study is carried out to help optimize the modules, and the corresponding results are shown in Table 2, where the ensemble empirical mode decomposition (EEMD), original variational mode decomposition (VMD), and the convolutional neural network (CNN), long short-term memory (LSTM) are chosen as competitors for the decomposition method and feature extraction, respectively. Moreover, paired t-tests with 10 samples of each model are implemented, and p-values are calculated to validate whether the performance differences between model variants are statistically significant. The p-values reflect that the performance improvements observed in ablation studies are significant.

**Table 2:** Comparison of ablation study. The deep model which consists of MOCOA-VMD and attention scheme achieves the best accuracy.

Number	Signal decomposition	Feature extraction	Best Accuracy(%)	P-value
1	None	CNN	79.90	$1.20\times 10^{-19}$
2	None	LSTM	81.95	$1.40\times 10^{-18}$
3	None	Attention scheme	83.63	$1.00\times 10^{-17}$
4	EEMD	CNN	88.20	$2.82\times 10^{-14}$
5	EEMD	LSTM	89.09	$4.19\times 10^{-13}$
6	EEMD	Attention scheme	88.60	$3.51\times 10^{-12}$
7	VMD	CNN	91.14	$1.35\times 10^{-09}$
8	VMD	LSTM	91.95	$1.89\times 10^{-08}$
9	VMD	Attention scheme	92.99	$5.49\times 10^{-05}$
10	MOCOA-VMD	CNN	93.56	$9.76\times 10^{-03}$
11	MOCOA-VMD	LSTM	93.98	$3.28\times 10^{-02}$
12	MOCOA-VMD	Attention scheme	94.46	1.00

It can be concluded that the attention scheme shows the best performance, followed by LSTM, which performs slightly worse, while CNN ranks last in terms of feature extraction. The attention scheme and LSTM generally exhibit superior performance in signal processing tasks due to their ability to handle temporal dependencies, sequence length flexibility, memory, and contextual information, adaptability to non-stationary data. Besides, the mode decomposition operation significantly improves the model performance. The performance of the model based on MOCOA-VMD reaches the best accuracy of 94.46%, followed by VMD, and EEMD last, which verifies the superiority of the deep attention model based on MOCOA-VMD proposed in this paper. Since the experimental values are adopted for setting the hyperparameters in the established deep attention model, to further improve the performance for classification, Bayesian optimization for hyperparameters will be studied and implemented in the next step.

### Bayesian optimization for hyperparameters

Bayesian optimization is a powerful technique that efficiently seeks an approximate optimal solution while minimizing the evaluation cost, which is especially advantageous for scenarios where the computation of fitness functions is resource-intensive or time-consuming [51]. Gaussian Process [52] (GP) is a commonly used non-parametric probabilistic surrogate model, widely applied in regression, classification, and many other domains that require inference of black-box function. Formally, a GP is specified by a mean function m:χ→ℝ and a semi-positive definite covariance function (or kernel) k:χ×χ→ℝ

$$f\left(x\right)\sim 𝒢𝒫\left(m\left(x\right),k\left(x,x^{\prime }\right)\right)$$ (39)

where m(x)=𝔼[f(x)] is the mean function, $k\left(x,x^{\prime }\right)=𝔼\left[\left(f\left(x\right)-m\left(x\right)\right)\left(f\left(x^{\prime }\right)-m\left(x^{\prime }\right)\right)\right]$ is the covariance function, which defines the covariance between the values of *f*(*x*) and $f(x^{\prime })$ at two points *x* and $x^{\prime }$.

Given training data $𝒟={\left\{\left(x_{i},y_{i}\right)\right\}}_{i=1}^{n}$ , where $y_{i}=f\left(x_{i}\right)$  +  ϵ and $\epsilon \sim 𝒩\left(0,\sigma_{n}^{2}\right)$ is Gaussian noise, GP regression is trying to predict the function value at new points x_*_. The joint distribution of the observed values *y* and the function values at the test points f_*_ is

$$\left(\begin{array}{c} y\\ f_{*} \end{array}\right)\sim 𝒩\left(\left(\begin{array}{c} m\left(x\right)\\ m\left(x_{*}\right) \end{array}\right),\left(\begin{array}{cc} K\left(x,x\right)+\sigma_{n}^{2}I & K\left(x,x_{*}\right)\\ K\left(x_{*},x\right) & K\left(x_{*},x_{*}\right) \end{array}\right)\right)$$ (40)

where K(x,x) is the covariance matrix of the training points, $K\left(x_{*},x\right)$ is the covariance matrix between test points and training points, $K\left(x_{*},x_{*}\right)$ is the covariance matrix of the test points. The predictive distribution at the test points x_*_ is Gaussian with mean and covariance

$$\begin{array}{ccc} \mu_{*} & = & K\left(x_{*},x\right){\left[K\left(x,x\right)+\sigma_{n}^{2}I\right]}^{-1}y\\ \Sigma_{*} & = & K\left(x_{*},x_{*}\right)-K\left(x_{*},x\right){\left[K\left(x,x\right)+\sigma_{n}^{2}I\right]}^{-1}K\left(x,x_{*}\right) \end{array}$$ (41)

where the mean $\mu_{*}$ provides the predicted value, while $\Sigma_{*}$ represents the uncertainty.

The acquisition function, often denoted as α, maps from the input space χ, the observation space ℝ, and the hyperparameter space Θ to the real number space, i.e., α:χ×ℝ×Θ→ℝ. The function constructs the posterior distribution obtained from the observed data set *D*_1:*t*_ and guides the selection of the next evaluation point *x*_*t* + 1_ by maximizing α at time step *t*

$$x_{t+1}=\underset{x\in \chi }{\max}\alpha_{t}\left(x;D_{1:t}\right)$$ (42)

One popular acquisition strategy is the Expected Improvement (EI), which is often used in conjunction with a Gaussian Process model. According to the Bayesian formula [53], EI at an acquisition point ${\text{y}}^{*}$ can be written as

$${EI}_{y^{*}}\left(x\right)=\int_{-\infty }^{y^{*}}\left(y^{*}-y\right)\frac{p\left(x|y\right)p\left(y\right)}{p\left(x\right)}dy$$ (43)

Given the GP’s predictive mean μ(x) and variance $\sigma^{2}(x)$ at a candidate point *x* and step *t*, the EI acquisition strategy based on the Gaussian process is specified as

$$\alpha_{t}\left(x;D_{1:t}\right)=\left\{ \begin{array}{cc} \left(v^{*}-\mu_{t}\left(x\right)\right)\phi \left(\frac{v^{*}-\mu_{t}\left(x\right)}{\sigma_{t}\left(x\right)}\right)+\sigma_{t}\left(x\right)\phi \left(\frac{v^{*}-\mu_{t}\left(x\right)}{\sigma_{t}\left(x\right)}\right), & \sigma_{t}\left(x\right)>0\\ 0, & \sigma_{t}\left(x\right)=0 \end{array}\right.$$ (44)

where ${\text{v}}^{*}$ is the best value observed so far, and ϕ(·) is the normal distribution probability density function.

The Tree-structured Parzen estimator [54] (TPE) models the objective function using two probability density functions

$$p\left(x|y\right)=\left\{ \begin{array}{cc} \ell \left(x\right) & \text{ if }y<y^{*}\\ g\left(x\right) & \text{ if }y\geq y^{*} \end{array}\right.$$ (45)

where ℓ(x) represents the density formed by using the observations {*x*^(*i*)^} such that the corresponding loss $\text{f}({\text{x}}^{\left(\text{i}\right)})$ was less than a threshold of ${\text{y}}^{*}$, g(x) is the density generated by collecting the remaining observations.

By constructing $\gamma =p\left(y<y^{*}\right)$, then $p\left(x\right)=\int_{\mathbb{R}}p\left(x|y\right)p\left(y\right)dy=\gamma \ell \left(x\right)$  +  (1−γ)g(x), so we have

$$\int_{-\infty }^{y^{*}}\left(y^{*}-y\right)p\left(x|y\right)p\left(y\right)dy=\gamma y^{*}\ell \left(x\right)-\ell \left(x\right)\int_{-\infty }^{y^{*}}p\left(y\right)dy$$ (46)

finally

$${EI}_{y^{*}}\left(x\right)=\frac{\gamma y^{*}\ell \left(x\right)-\ell \left(x\right)\int_{-\infty }^{y^{*}}p\left(y\right)dy}{\gamma \ell \left(x\right)+\left(1-\gamma \right)g\left(x\right)}\propto {\left(\gamma +\frac{g\left(x\right)}{\ell \left(x\right)}\left(1-\gamma \right)\right)}^{-1}$$ (47)

EI’s goal is to balance exploration and exploitation by taking into account both the predicted mean and uncertainty of the model. Consequently, to fully optimize point X, it is essential to maximize the function ℓ(x) and minimize the function *g*(*x*). During each iteration, the TPE algorithm returns the candidate ${\text{x}}^{*}$ with the highest expected improvement.

In Bayesian optimization, batch size, learning rate, number of epochs, and momentum are determined as key parameters to optimize. Batch size defines the number of training samples used in one iteration of training a neural network, which calls for a compromise between computational efficiency and model convergence behavior. While trying to minimize the loss function, the learning rate controls the step size at each iteration. The number of epochs specifies how many times the learning algorithm will work through the entire training dataset, which directly impacts the model’s performance and generalization ability. Momentum is a technique used in the optimization process of training neural networks to accelerate the gradient vectors in the right directions, thus leading to faster convergence and helping to smooth out the gradients and avoid getting stuck in local minima.

**Fig 13:**
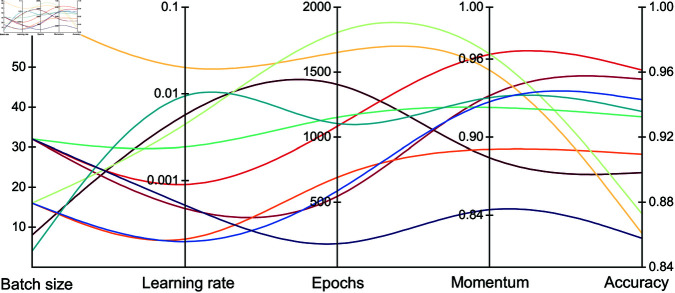
Bayesian optimization results of key hyperparameters.

Referring to the general experience of fine-tuning a deep model, the ranges of values for the key design parameters are determined as batch size {4,8,16,32,64}, learning ate [0.0001,0.1]∩ℝ, epochs [200,2000]∩ℤ, momentum [0.8,0.99]∩ℝ. The design space is composed of all the combinations of the above key parameters. The Gaussian Process surrogate model and TPE algorithm are put into operation for the deep attention models that demand high evaluation costs. The results of the top 10 performance combinations are shown in Fig 13. The best accuracy of 96.11% occurs with a combination of batch size 32, learning rate 0.00092, epochs 1070, and momentum 0.966, which is higher by 1.65% than the model before optimization.

**Fig 14:**
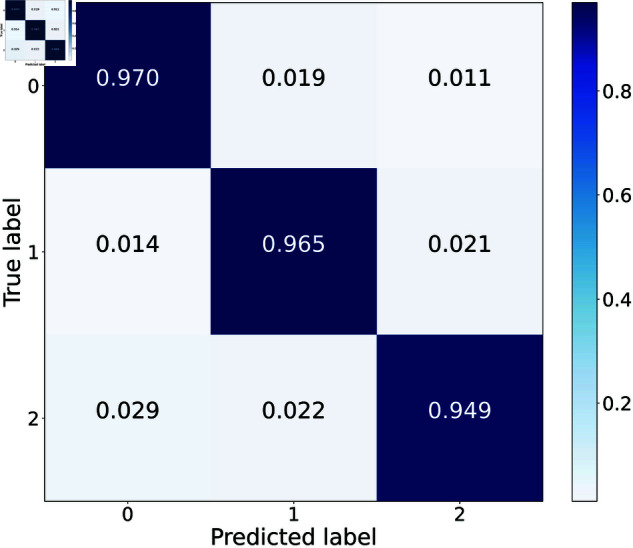
Confusion matrix.

The performance measurement tool confusion matrix is utilized to evaluate the accuracy of a classification model, as shown in Fig 14. It can be seen that the proposed model achieves excellent success in the classification of the different patterns of ECG signals, with accuracies up to 97.06% and 96.47% for signal 0 and signal 1, respectively, and slightly lower at 94.91% for signal 2. We speculate that it is possibly due to the varying characteristics modeled by PDEs for arrhythmia ECG patterns.

### Model validation on MIT-BIH database

To assess the robustness and generalizability of the proposed model, we utilize the well-known MIT-BIH Arrhythmia Database for real-world ECG signal analysis. This database, provided by PhysioNet, includes annotated ECG recordings from 47 patients, totaling approximately 25 hours of recording time. Each record contains two lead ECG signals (MLII and V1) sampled at 360 Hz with 12-bit resolution. The comprehensive annotations facilitate the evaluation of our algorithm’s performance in detecting various arrhythmias.

**Fig 15:**
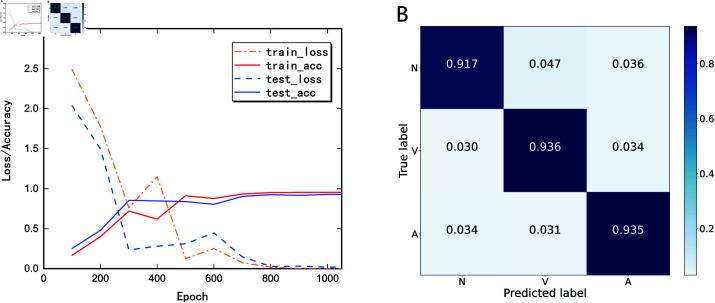
Performance validation on the MIT-BIH database. (A) Training state. (B) Confusion matrix.

Three different type of ECGs are chosen for model validation, i.e., 75052 samples of normal sinus beat (N), 7130 samples of premature ventricular contraction (V) and 2546 samples of premature atrial contraction (A). The dataset was divided into 80% for training and 20% for testing. The results of the performance validation conducted on the MIT-BIH Arrhythmia Database after 1000 epochs are shown in Fig 15. The training state curve in Fig 15(A) shows that the accuracy reaches 95.46% on the training set and 92.87% on the test set. The confusion matrix in Fig 15(B) reveals that the model performs less accurately on normal sinus beats compared to premature ventricular contractions and premature atrial contractions, which may necessitate further investigations in the future.

The experiment results are compared with other related studies based on the MIT-BIH database, as shown in Table 3. It can be seen that the deep model proposed in this paper performs well but needs to be further improved in future studies. Integrating different deep modules, such as CNN, RNN and LSTM for feature extraction may be a promising direction.

**Table 3:** Performance comparison with other papers.

Paper	Number of classes	Models	Accuracy(%)
[8]	5	CNN+LSTM	97.15
[13]	14	ELM-RNN	96.41
[14]	5	SMOTE+CNN	92.73
[15]	3	random forest	88.70
this paper	3	VMD+attention scheme	92.87

In summary, the deep VMD-attention network based on the multi-objective crayfish optimization algorithm in this paper is highly effective for classification tasks and is believed to have the potential to be utilized in other signal processing and classification issues.

## Conclusion

This paper proposes a novel deep attention model for arrhythmia signal processing based on multi-objective crayfish optimization algorithmic variational mode decomposition. The main conclusions are as follows.

1. A finite element model of the human heart based on the Hodgkin-Huxley model was established for cardiac electrophysiology simulation, and the ECG signals were obtained from the FEM results of representative points. Two distinct types of arrhythmia, characterized by major anomalies of parameters *I* and *h* in the HH model, were examined in detail, and the corresponding ECG signals were also obtained through simulation. The typical synthetic signals from the three models with built-in Gaussian noise, were prepared for signal decomposition and classification.
2. A variational mode decomposition technique for ECG signal processing based on a multi-objective crayfish optimization algorithm (MOCOA-VMD) was proposed. A multi-objective optimization method based on non-dominated sorting was incorporated into the crayfish optimization algorithm to search for the optimal *K* and α in VMD processing, and the spectral kurtosis and KL divergence were determined as the indicators for decomposition. The Pareto optimal front was generated by MOCOA and the intrinsic mode functions of VMD with the best combination of *K* and α were obtained.
3. A deep VMD-attention model based on MOCOA was constructed for ECG signal classification. To verify the effectiveness of the proposed signal decomposition method and deep attention modules, an ablation study was implemented. The performance of the model based on MOCOA-VMD achieves the best accuracy of 94.46%, much higher than the model constructed by modules of EEMD, VMD, CNN and LSTM. Moreover, Bayesian optimization was carried out to fine-tune the hyperparameters batch size, learning rate, epochs, and momentum. The best accuracy of the deep attention model after TPE optimization reaches 96.11%, which is 1.65% higher than the original model. The real-world ECG signals from the MIT-BIH arrhythmia database were utilized for further validation, and the accuracy reached 95.46% on the training set and 92.87% on the test set, proving the robustness and generalizability of the proposed model.

In conclusion, the deep VMD-attention network based on the HH model and MOCOA has demonstrated significant success in the classification of ECG signals. The method proposed in this paper shows substantial potential for both mathematical modeling and practical applications. Looking forward, on the one hand, the VMD-attention network needs to be further optimized to improve performance, and the mathematical models for different arrhythmias’ electrophysiology are to be established. On the other hand, we anticipate expanding this ECG signal modeling and processing strategy to various other biological time-dependent and non-stationary signal processing domains, such as electroencephalogram (EEG) signals, respiratory signals, blood pressure signals, and electrooculography (EOG).
